# Front-line intraperitoneal versus intravenous chemotherapy in stage III-IV epithelial ovarian, tubal, and peritoneal cancer with minimal residual disease: a competing risk analysis

**DOI:** 10.1186/s12885-016-2279-0

**Published:** 2016-03-17

**Authors:** Yen-Hou Chang, Wai-Hou Li, Yi Chang, Chia-Wen Peng, Ching-Hsuan Cheng, Wei-Pin Chang, Chi-Mu Chuang

**Affiliations:** Institute of Clinical Medicine, School of Medicine, National Yang-Ming University, Taipei, Taiwan; Division of Gynecologic Oncology, Department of Obstetrics and Gynecology, Taipei Veterans General Hospital, Taipei, Taiwan; Institute of Public Health, School of Medicine, National Yang-Ming University, Taipei, Taiwan; School of Health Care Administration, Taipei Medical University, Taipei, Taiwan; Department of Obstetrics and Gynecology, Cardinal Tien Hospital, Taipei, Taiwan; Department of Obstetrics and Gynecology, Fu-Jen Catholic University, Taipei, Taiwan; 250 Wuxing Street, Taipei City, 110 Taiwan; No.155, Sec.2, Linong Street, Taipei, 11217 Taiwan

**Keywords:** Competing risk, Ovarian cancer, Intraperitoneal chemotherapy, Propensity score, Subdistribution hazard ratio

## Abstract

**Background:**

In the analysis of survival data for cancer patients, the problem of competing risks is often ignored. Competing risks have been recognized as a special case of time-to-event analysis. The conventional techniques for time-to-event analysis applied in the presence of competing risks often give biased or uninterpretable results.

**Methods:**

Using a prospectively collected administrative health care database in a single institution, we identified patients diagnosed with stage III or IV primary epithelial ovarian, tubal, and peritoneal cancers with minimal residual disease after primary cytoreductive surgery between 1995 and 2012. Here, we sought to evaluate whether intraperitoneal chemotherapy outperforms intravenous chemotherapy in the presence of competing risks. Unadjusted and multivariable subdistribution hazards models were applied to this database with two types of competing risks (cancer-specific mortality and other-cause mortality) coded to measure the relative effects of intraperitoneal chemotherapy.

**Results:**

A total of 1263 patients were recruited as the initial cohort. After propensity score matching, 381 patients in each arm entered into final competing risk analysis. Cumulative incidence estimates for cancer-specific mortality were statistically significantly lower (*p* = 0.017, Gray test) in patients receiving intraperitoneal chemotherapy (5-year estimates, 34.5 %; 95 % confidence interval [CI], 29.5–39.6 %, and 10-year estimates, 60.7 %; 95 % CI, 52.2–68.0 %) versus intravenous chemotherapy (5-year estimates, 41.3 %; 95 % CI, 36.2–46.3 %, and 10-year estimates, 67.5 %, 95 % CI, 61.6–72.7 %). In subdistribution hazards analysis, for cancer-specific mortality, intraperitoneal chemotherapy outperforms intravenous chemotherapy (Subdistribution hazard ratio, 0.82; 95 % CI, 0.70–0.96) after correcting other covariates.

**Conclusions:**

In conclusion, results from this comparative effectiveness study provide supportive evidence for previous published randomized trials that intraperitoneal chemotherapy outperforms intravenous chemotherapy even eliminating the confounding of competing risks. We suggest that implementation of competing risk analysis should be highly considered for the investigation of cancer patients who have medium to long-term follow-up period.

**Electronic supplementary material:**

The online version of this article (doi:10.1186/s12885-016-2279-0) contains supplementary material, which is available to authorized users.

## Background

Ovarian cancer is the sixth most common tumor in women. More than 200,000 new cases are diagnosed each year worldwide. Each year, it constitutes 4 % of all cancers diagnosed in women, and there are 6.6 new cases per 100,000 women per year [1]. Epithelial ovarian cancer takes the predominant 90 % of cases among ovarian cancer patients.

Epithelial ovarian cancer typically spreads by intraperitoneal seeding or direct invasion or through the lymphatic and vascular circulation. Among the spreading routes, peritoneal spreading is the most common route of dissemination, and stage III disease is associated at best with a 5-year survival rate of approximately 32–47 % [2]. Therefore, it is reasonable to consider the intraperitoneal chemotherapy for this disease entity [3]. Intraperitoneal chemotherapy has been investigated for several decades, and there have been three large-scale randomized trials conducted in the US, all of which showed overall and/or progression-free survival benefit [4–6].

Most published studies for ovarian cancer use the time to some disease events as their primary outcome and hence, statistical methods developed for survival data are usually applied. Established methods for estimating and modelling these include the Kaplan–Meier estimator of the survival function and the Cox proportional hazards model for the hazard function [7, 8] An important assumption of these established survival analytical methods is that censoring is ‘independent’ [9]. However, in some cases, several causes of failure are possible but the occurrence of one event precludes the occurrence of the other events (i.e., when failures are different causes of death, only the first one can be observed). This situation is known as competing risks. In a competing risk situation, standard techniques for survival analysis may lead to incorrect and biased results [10, 11]. In usual condition, ovarian cancer often presents a protracted disease course, and it is not uncommon to see a patient dies of other causes (e.g., heart failure and stroke), which precludes the occurrence of cancer-specific death.

In the current work, we conducted a competing risk analysis to investigate the therapeutic effects of intraperitoneal chemotherapy on stage III-IV epithelial ovarian, tubal, and peritoneal cancer with minimal residual disease using an administrative health care database constructed in a single tertiary care institution.

## Methods

### Study population

The study entailed a retrospective analysis of prospectively collected demographic, tumor profile, treatment, and comorbidity data, linking electronic data sources, including the cancer registry, administrative and clinical databases, and surgery records of consecutive patients with stage III or IV primary epithelial ovarian, tubal, and peritoneal cancers with minimal residual disease after primary cytoreductive surgery between January 1995 and December 2012. The standard patient informed consent for retrieval of personal information at the institution included an emphasized section describing the purpose of the study. This study was approved by the Institutional Review Board of Taipei Veterans General Hospital. The procedures used in this study were in accordance with the guidelines of the Helsinki Declaration on human experimentation.

### Primary cytoreductive surgery and front-line chemotherapy

The standard cytoreductive surgery included total hysterectomy, bilateral salpingo-oophorectomy, infracolic omentectomy, and cytoreduction of all tumor nodules to a size of 1 cm or less in the greatest dimension.

Courses of front-line chemotherapy were repeated every 3 weeks for a total of six cycles, provided the serum creatinine concentration was less than or equal to 2.0 mg/dl, the white-cell count was higher than 3000/mm^3^, and the platelet count was higher than 80,000/mm^3^. Dosing schedules for intraperitoneal chemotherapy were either platinum-based or taxane-based. For platinum-based intraperitoneal chemotherapy, cisplatin at 100 mg/m^2^ or carboplatin at AUC 5 or 6 via Tenckhoff tubes was administered. For taxane-based intraperitoneal chemotherapy, protocol for GOG 172 was followed.^6^

For intravenous chemotherapy, both taxane (175 mg/m^2^) plus cisplatin (dosed at 50 mg/m^2^) or carboplatin (AUC = 5 or 6) were administered every 3 weeks for a total of six cycles.

In each cycle of chemotherapy, patients received physical examination, complete blood count, biochemical profiles, CA-125, and 24-h urine collection for measurement of clearance of creatinine. In the absence of clinical evidence of tumor progression, tumor evaluation by image studies including chest film, whole abdominal sonography, and CT scan (or MRI) was performed after six cycles of chemotherapy.

### Statistical analyses

Continuous variables are presented as mean (± standard deviations) and were compared using Student’s unpaired t-test. Categorical variables are presented as counts and percentages and were compared with the χ^2^ test when appropriate (expected frequency > 5). Otherwise, Fisher’s exact test was used. Overall survival duration was calculated from disease diagnosis to event occurrence or last follow-up. Patients who succumbed to cancer-related death were classified as cancer-specific mortality (CSM), while patients who succumbed to other causes were classified as other-cause mortality (OCM).

Our statistical analyses consisted of two steps. In the first step, we attempted to adjust for the selection bias inherent in observational data by applying propensity score matching to balance the measured covariates between the intravenous and intraperitoneal chemotherapy group. The propensity score is a summary confounder score that is modeled using the exposure or treatment as the dependent variable [12]. The propensity to receive intraperitoneal chemotherapy was calculated using a multivariable logistic regression model that adjusted to age at diagnosis, FIGO stage, tumor grade, histology, Charlson comorbidity index, and GOG performance score. We used the nearest neighbor matching with a caliper width of 0.2 of the standardized deviation of the logit to match cases. This optimizes the matching with minimal residual bias and highest precision [13]. Covariate balance was evaluated using standardized differences of means (SDM), with SDM of < 0.1 (corresponding to <10 % difference between the arms) indicative of acceptable balance [14].

In the second step of analyses, competing risks analyses were conducted based on the propensity score-matched cohort. The crude cumulative incidence function were estimated for each type of competing risk using the method of Kalbfleisch and Prentice [15, 16] Cumulative incidence plots were used to graphically depict CSM and OCM rates. Statistical significance of differences in survival rates was assessed with the Gray test [17] Furthermore, we investigated differences in each cause of mortality (for CSM and OCM, respectively) using subdistribution hazard ratios estimated through Fine and Gray proportional hazards regression [18] Censoring time was set at December 31, 2012. The dataset supporting the conclusions of this article is listed in Additional file 1.

Finally, we performed a sensitivity analysis based on different sample size when extreme values of the propensity score were trimmed at different levels - a procedure shown to partly compensate for unobserved confounding [19].

All tests were performed two sided at the 5 % significance level. Statistical analyses were performed with IBM SPSS Statistics (version 20.0, IBM, Armonk, NY), and R software (version 2.15, R Foundation for Statistical Computing, Vienna, Austria) using the cmprsk, survival, and Matching packages [20].

## Results

A total of 1263 patients were recruited as the initial cohort. The patients’ baseline clinical characteristics of the initial cohort are summarized in the left part of Table 1. In terms of standardized difference of means, there were significant differences in age, stage, grade, histologic subtype, proportion of front-line regimen, GOG performance status, and Charlson comorbidity score. However, after propensity score-matching (summarized at right part of Table 1), the standardized difference of means in all the baseline clinical characteristics was less than 10 %, which indicates a high degree of similarity in the distribution of these covariates, with equal number of 381 patients receiving intravenous chemotherapy and intraperitoneal chemotherapy, respectively. The propensity score-matched cohort forms the basis for the following analyses. The median follow-up time for this cohort was 8.4 years.

**Table 1:** Descriptive characteristics of patients treated with intravenous vs. intraperitoneal chemotherapy for stage III-IV epithelial ovarian, tubal, and peritoneal cancer with minimal residual disease between 1995 and 2012 (*n* = 1263)

	Initial cohort (*n* = 1263)			Propensity score-matched cohort (*n* = 548)		
	intravenous	intraperitoneal	SDM	intravenous	intraperitoneal	SDM^a^
	*n* = 847	*n* = 416		*n* = 381	*n* = 381	
Characteristics	(71.4 %)	(28.6 %)		(50 %)	(50 %)	
Age (y, mean)	54.3	52.6	0.017	52.7	52.6	0.002
Stage (%)						
III	671 (90.1 %)	267 (89.6 %)	0.014	254 (92.7 %)	252 (92.0 %)	0.013
IV	74 (9.9 %)	31 (10.4 %)	−0.020	20 (7.3 %)	22 (8.0 %)	−0.021
Grade (%)						
1	119 (16.0 %)	43 (14.4 %)	0.011	33 (12.0 %)	37 (13.5 %)	−0.021
2	429 (57.6 %)	180 (60.4 %)	−0.008	178 (65.0 %)	176 (64.2 %)	0.017
3	197 (26.4 %)	75 (25.2 %)	0.009	63 (23.0 %)	61 (22.2 %)	0.011
Histologic subtype (%)						
Serous	371 (49.8 %)	145 (48.7 %)	0.016	142 (51.8 %)	140 (51.1 %)	0.006
Mucinous	75 (10.1 %)	26 (8.7 %)	0.014	21 (7.7 %)	24 (8.8 %)	−0.016
Clear cell	109 (14.6 %)	41 (13.8 %)	0.007	41 (15.0 %)	38 (13.9 %)	0.014
Endometrioid	83 (11.1 %)	45 (15.1 %)	−0.037	48 (17.5 %)	45 (16.4 %)	0.010
Mixed or others^a^	107 (14.4 %)	41 (13.8 %)	0.009	22 (8.0 %)	27 (9.9 %)	−0.019
Front-line regimen (%)						
Paclitaxel based	661 (88.8 %)	259 (81.2 %)	0.074	245 (89.4 %)	248 (90.5 %)	−0.008
Not paclitaxel based	84 (11.3 %)	39 (13.1 %)	−0.017	29 (10.6 %)	26 (9.5 %)	0.009
Consolidation chemotherapy (%)						
No	622 (83.5 %)	242 (83.9 %)	−0.007	202 (73.7 %)	203 (74.1 %)	−0.005
Yes	123 (16.5 %)	56 (16.1 %)	0.008	72 (26.3 %)	71 (25.9 %)	0.006
GOG performance status						
≤ 1	649 (87.1 %)	250 (83.9 %)	0.026	241 (88.0 %)	239 (87.2 %)	0.003
≥ 2	96 (12.9 %)	48 (16.1 %)	−0.027	33 (12.0 %)	35 (12.8 %)	−0.006
Charlson comorbidity index (%)						
0	637 (85.5 %)	231 (77.5 %)	0.103	223 (81.4 %)	219 (80.0 %)	0.008
≥ 1	108 (14.5 %)	67 (22.5 %)	−0.146	51 (18.6 %)	55 (20.0 %)	−0.014

*Abbreviations* *SDM* standardized difference of means

^a^Others histology include: undifferentiated and unclassified epithelial carcinoma

**Fig. 1:**
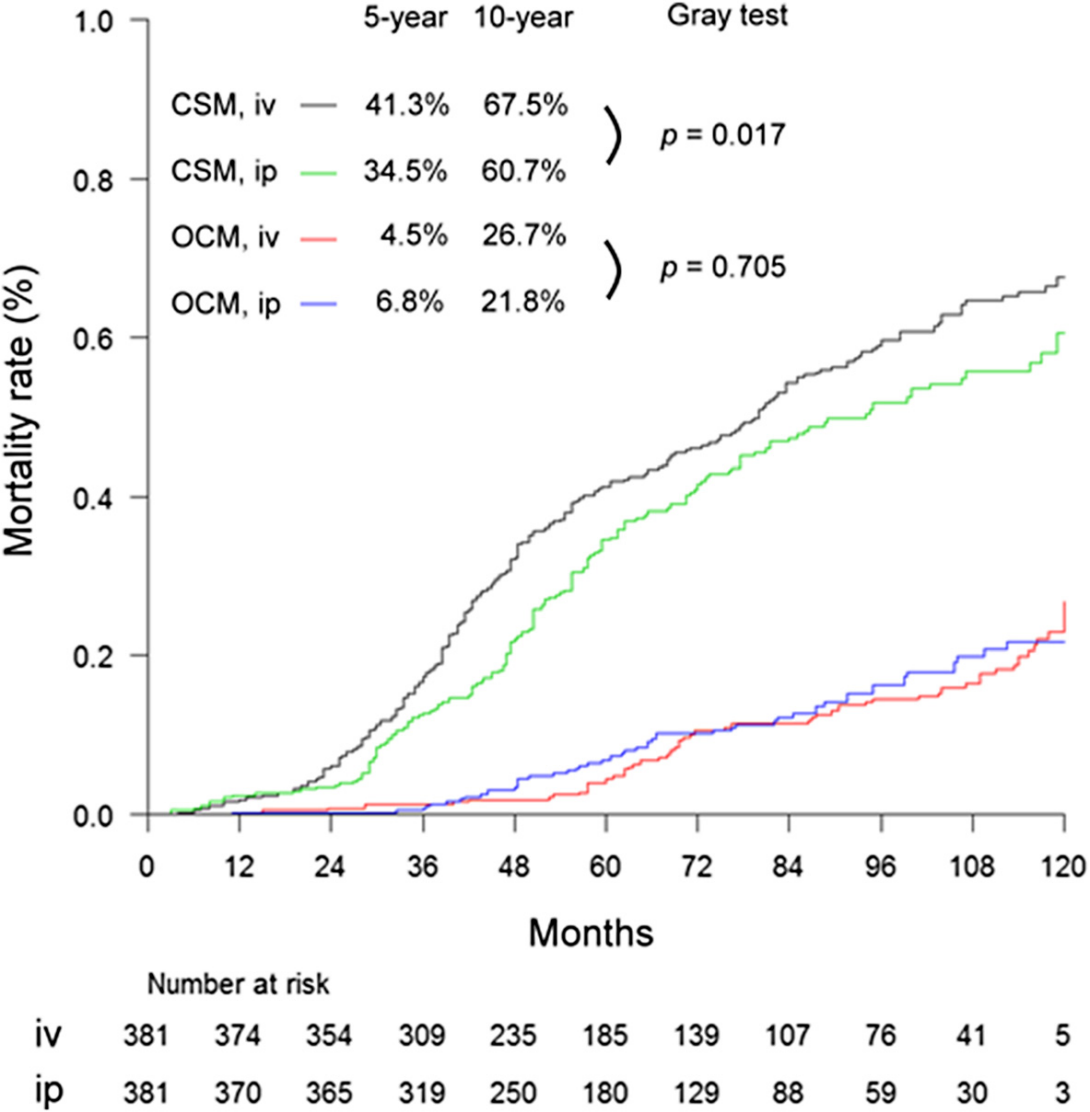
Curves for cumulative incidence function stratified by CSM and OCM, and further sub-stratified by iv or ip chemotherapy. Abbreviations: iv, intravenous chemotherapy; ip, intraperitoneal chemotherapy

Figure 1 shows the estimates and curves of cumulative incidence function stratified by CSM and OCM, and sub-stratified by type of chemotherapy (intravenous vs. intraperitoneal chemotherapy). Cumulative incidence estimates for CSM were statistically significantly lower (*p* = 0.017, Gray test) in patients receiving intraperitoneal chemotherapy (5-year estimates, 34.5 %; 95 % confidence interval [CI], 29.5–39.6 %, and 10-year estimates, 60.7 %; 95 % CI, 52.2–68.0 %) versus intravenous chemotherapy (5-year estimates, 41.3 %; 95 % CI, 36.2–46.3 %, and 10-year estimates, 67.5 %, 95 % CI, 61.6–72.7 %). In contrast, cumulative incidence estimates for OCM were comparable (*p* = 0.705, Gray test) between patients receiving intraperitoneal chemotherapy (5-year estimates, 6.8 %; 95 % CI, 4.4–9.8 %, and 10-year estimates, 21.8 %, 95 % CI, 16.5–27.5 %) and receiving intravenous chemotherapy (5-year estimate, 4.5 %; 95 % CI, 2.9–7.6 %, and 10-year estimate, 26.7 %; 95 % CI, 20.6–32.6 %).

**Fig. 2:**
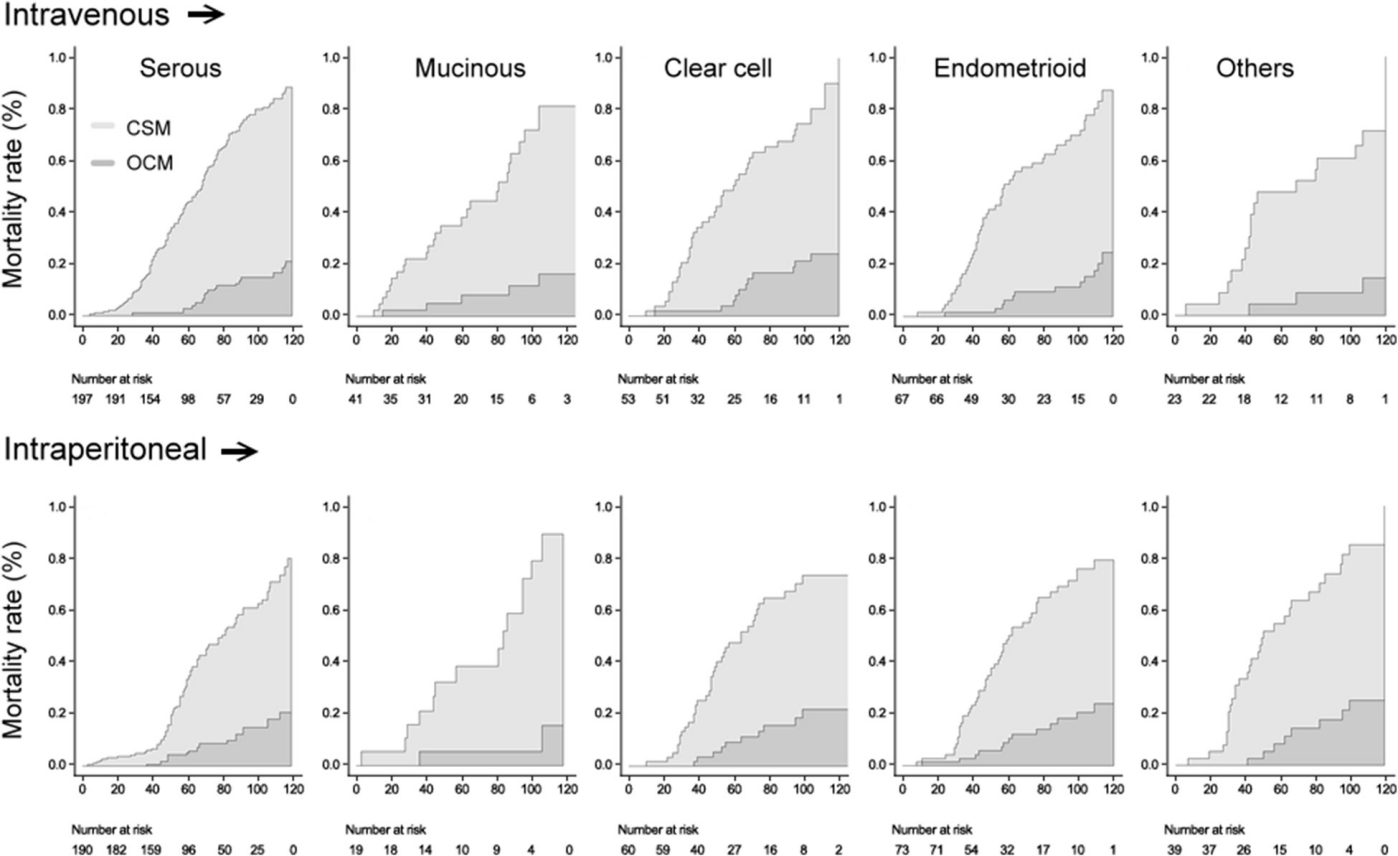
Curves for cumulative incidence function for each histologic subtype

Because ovarian cancer is now considered as a heterogeneous disease in which histologic phenotypes correlate with distinct genetic events, therefore we present cumulative incidence estimates for each histologic subtypes. For each histologic subtype, there remains a significant difference for CSM between intravenous chemotherapy and intraperitoneal chemotherapy. However, for OCM, there is no difference between intravenous chemotherapy and intraperitoneal chemotherapy (Fig. 2).

In conventional survival analysis, application of Cox proportional hazards regression uses multivariable hazard ratios can account for confounding. Nonetheless, Cox regression may lead to biased effect estimates in the presence of competing risks [21]. Hence, we next conducted unadjusted and adjusted regression modelling proposed by Fine and Gray in the presence of competing risks [18]. Unadjusted and adjusted subdistribution hazard ratios for CSM and OCM are given in Table 2. For CSM, several factors demonstrate statistically significant which include stage, grade, histology, and type of chemotherapy in the univariable analysis. However, after multivariable regression modelling, only some factors remain as an independent risk factor, including stage (IV vs. III, subdistribution hazard ratio [SDHR], 2.66; 95 % CI, 1.47–4.81]), grade (grade 3 vs. grade 1, SDHR, 1.89; 95 % CI, 1.04–3.43), histology (clear cell vs. serous, SDHR, 1.68; 95 % CI, 1.14–2.48, and others vs. serous, SDHR, 2.36; 95 % CI, 1.67–3.34), and type of chemotherapy (intraperitoneal vs. intravenous chemotherapy, SDHR, 0.82; 95 % CI, 0.70–0.96). Hence intraperitoneal chemotherapy remains a significant factor after multivariable regression modelling in competing risk analysis.

**Table 2:** Results of the subdistribution hazard regression (Fine and Gray) models for both type of failures

	Unadjusted		Adjusted^c^	
Patient categories	SDHR (95 % CI)^b^	*P* value	SDHR (95 % CI)^b^	*P* value
CSM				
Age^a^	0.96 (0.75–1.23)	0.121	0.95 (0.67–1.35)	0.132
Stage				
III	1		1	
IV	2.43 (1.58–3.73)	0.026	2.66 (1.47–4.81)	0.021
Grade				
1	1		1	
2	1.24 (0.97–1.59)	0.037	1.36 (0.99–1.94)	0.088
3	1.57 (1.03–2.39)	0.024	1.89 (1.04–3.43)	0.031
Histology				
Serous	1		1	
Mucinous	0.93 (0.82–1.05)	0.095	0.95 (0.79–1.14)	0.118
Clear cell	1.43 (1.02–2.01)	0.036	1.68 (1.14–2.48)	0.029
Endometrioid	0.96 (0.82–1.12)	0.102	0.98 (0.89–1.07)	0.095
Others^d^	2.17 (1.65–2.85)	0.028	2.36 (1.67–3.34)	0.025
Type of chemotherapy				
intravenous	1		1	
intraperitoneal	0.78 (0.67–0.91)	0.029	0.82 (0.70–0.96)	0.031
GOG performance status				
≤ 1	1		1	
≥ 2	0.83 (0.68–1.02)	0.075	0.92 (0.81–1.06)	0.079
Charlson comorbidity index				
0	1		1	
≥ 1	0.84 (0.68–1.04)	0.083	0.88 (0.70–1.09)	0.089
OCM				
Age^a^	0.95 (0.91–0.99)	0.038	0.92 (0.87–0.97)	0.017
Stage				
III	1		1	
IV	0.92 (0.77–1.11)	0.337	0.87 (0.69–1.04)	0.214
Grade				
1	1		1	
2	0.87 (0.65–1.16)	0.298	0.91 (0.64–1.29)	0.348
3	0.92 (0.59–1.47)	0.361	0.87 (0.55–1.37)	0.229
Histology				
Serous	1		1	
Mucinous	1.06 (0.89–1.26)	0.139	1.07 (0.85–1.35)	0.168
Clear cell	1.01 (0.76–1.18)	0.106	1.05 (0.86–1.28)	0.143
Endometrioid	0.95 (0.82–1.10)	0.295	0.98 (0.79–1.21)	0.315
Others^d^	0.91 (0.81–1.02)	0.082	0.89 (0.75–1.05)	0.091
Type of chemotherapy				
Intravenous	1		1	
Intraperitoneal	0.89 (0.73–1.22)	0.127	0.94 (0.81–1.09)	0.115
GOG performance status				
≤ 1	1		1	
≥ 2	1.05 (0.84–1.38)	0.229	1.03 (0.82–1.36)	0.226
Charlson comorbidity index				
0	1		1	
≥ 1	0.77 (0.65–0.92)	0.022	0.79 (0.66–0.94)	0.027

*Abbreviations CSM* cancer-specific mortality, *OCM* other-cause mortality, *CI* confidence interval

^a^per 10 year increment in age

^b^SDHR denotes subdistribution hazards ratio obtained by Fine-Gray model. CI, confidence interval

^c^Adjusted for the following factors: age, stage, nuclear grade, histologic subtype, type of chemotherapy

^d^“Others” histology includes mixed type, undifferentiated, and carcinosarcoma

For OCM, two factors demonstrate statistically significant which include age and Charlson comorbidity index in the univariable analysis. After multivariable regression modelling, these two factors still remain as an independent factor: age (per 10 years increment, SDHR, 092; 95 % CI, 0.87–0.97) and Charlson comorbidity index (≥1 vs. 0, SDHR, 0.79; 95 % CI, 0.66–0.94).

Lastly we conducted a sensitivity analysis for CSM. Consistent results of subdistribution hazard ratio of intraperitoneal chemotherapy vs. intravenous chemotherapy were obtained when extreme values of the propensity score were trimmed at different levels − a procedure shown to partly compensate for unobserved confounding (Table 3).

**Table 3:** Sensitivity analysis of estimated subdistribution hazard ratio for CSM by removing a specific percent of cases at the extremes of propensity score^a^

% of PS values trimmed	N^c^	SDHR	95 % CI	*P*
0^b^	548	0.82	0.70–0.96	0.014
1	514	0.81	0.79–0.81	<0.001
3	472	0.79	0.67–0.93	0.005
5	428	0.79	0.66–0.92	0.004
10	364	0.77	0.64–0.93	0.006
15	248	0.75	0.60–0.94	0.012
20	196	0.72	0.53–0.98	0.036
25	128	0.76	0.61–0.95	0.014

*Abbreviations* *CI* confidence interval, *CSM* cancer-specific mortality, *PS* propensity score, *SDHR* subdistribution hazards ratio

^a^The results demonstrates stability of subdistribution hazard ratio across the iterations

^b^The "0 %" trim indicates limiting the analysis to the region of propensity score overlap

^c^N indicates number of patients remaining in the analysis

## Discussion

This competing risks analyses based on a single-institution database of patients with stage III-IV epithelial ovarian, tubal, and peritoneal cancer with minimal residual disease demonstrate better survival outcome treated by intraperitoneal chemotherapy compared to intravenous chemotherapy with respect to CSM. Intraperitoneal chemotherapy has been investigated for a long time, and based on the positive results from a meta-analysis conducted by National Cancer Institute (NCI) and the Gynecologic Oncology Group, the NCI has released a clinical announcement encouraging the gynecological oncology community to consider intraperitoneal chemotherapy using cisplatin as the standard treatment for advanced ovarian cancer patients in whom the residual disease were debulked to 1 cm or less [22]. The results of the current work can further consolidate the role of intraperitoneal chemotherapy in the treatment of advanced ovarian cancer.

Competing risks methods are common in biomedical research, particularly in cancer, where the need to deal with multiple potential outcomes is nearly ubiquitous. In fact, the concept of competing risks within clinical research was first introduced in the field of oncology [23]. As treatment for cancer produced prolonged survival times, it became important to consider not only the effects of treatment on cancer-free survival, but also how competing risks, such as mortality from unrelated causes, might impact treatment decisions. For example, competing risks methods have been applied in the analyses of risk factors for breast cancer, either at the screening or treatment stage that may potentially impact the decision making [24–27].

Traditionally, when predict the unadjusted probability of a certain event of interest to occur, one can use the Kaplan–Meier (KM) method. However, in the presence of competing risks, using the KM method is problematic. This method can handle only one single event at one time, and all other events are treated as censored observations. Further, the complement of the KM estimate (1 − KM) is interpreted as the cumulative probability of the event of interest in a hypothetical world where no subject would experience the competing event. This kind of interpretation is not realistic in clinical practice [28].

While for the adjusted analysis of competing risk, Fine and Gray proposed a regression modeling applied directly on a cumulative incidence function for particular use in the competing risk analysis. For any event type, this approach focuses on the hazard associated with the cumulative incidence function.

Several limitations merit consideration in the current work. First, the composition of the initial cohort may be influenced by referral bias because of the monocentric design. The index hospital is a tertiary referral center with more patients harboring co-morbidities. Thus, the results of the present study may not be generalizable to the general population. Second, lack of laboratory data may influence the robustness of the results. For example, serum cancer antigen 125 (CA125) is widely used in ovarian cancer to monitor the effectiveness of therapy both in first line and recurrence [29, 30]. Lack of inclusion of CA125 into the calculation of propensity-score may `bias our results. Third, limitations in patient-level data collection were also present. For example, socioeconomic status have been found to be associated with cancer mortality [31] yet, our database did not capture this important factor which potentially affects the reliability of both propensity-score and competing risk modelling. Fourth, the protocol of follow-up was not strictly defined and censoring recordings may not be reflective of the true status. Fifth, the current analysis have not been validated with an external dataset which limit its clinical application.

Competing risks analyses allow disentangling the contribution of an intervention (e.g., intraperitoneal chemotherapy) on each event type separately. Intraperitoneal chemotherapy clearly shows therapeutic benefit in terms of CSM, but not OCM in the current work. In addition to intraperitoneal chemotherapy, stage IV (vs. III), grade 3 (vs. grade 1), and clear cell subtype (vs. serous subtype) also demonstrate as significant risk factors. Clear cell carcinoma accounts for 4 to 12 % of epithelial ovarian cancer in Western countries. Compared to serous adenocarcinoma, clear cell carcinoma is relatively resistant to conventional platinum, or taxane-based chemotherapy which is associated with its poor prognosis [32]. The results of our work suggest that novel therapy should be developed for clear cell carcinoma in order to improve survival outcome.

From published reports, the mean or median age for ovarian cancer is between 55 and 60 years which implies that a significant proportion of patients are facing the problem of aging. During the course of treatment for cancer, the increased age and co-morbidities of older patients occasionally lead to death by the occurrence of competing events (e.g., stroke, infection, and accident). Clinical studies are often faced with the difficult problem of how to account for participants who die without experiencing the study outcome of interest. Conventional approaches to describe risk of disease like Kaplan-Meier survival analysis and Cox proportional hazards regression can overestimate risk of disease by failing to account for the competing risk of death [33].

## Conclusions

In conclusion, results from this competing risk analysis provide supportive evidence for previous published randomized trials that intraperitoneal chemotherapy outperforms intravenous chemotherapy. We propose that implementation of competing risk analysis should be highly considered for the investigation of ovarian cancer patients who have medium to long-term follow-up period.

## Additional file

Additional file 1: Dataset for computing competing risks. (CSV 18 kb)

## Abbreviations

CSM: cancer-specific mortality
IP: intraperitoneal
IV: intravenous
OCM: other-cause mortality
